# Mental causation: an evolutionary perspective

**DOI:** 10.3389/fpsyg.2024.1394669

**Published:** 2024-04-29

**Authors:** Thurston Lacalli

**Affiliations:** Department of Biology, University of Victoria, Victoria, BC, Canada

**Keywords:** animal consciousness, epiphenomenalism, agency, free will, EM field, memory theories of consciousness

## Abstract

The relationship between consciousness and individual agency is examined from a bottom-up evolutionary perspective, an approach somewhat different from other ways of dealing with the issue, but one relevant to the question of animal consciousness. Two ways are identified that would decouple the two, allowing consciousness of a limited kind to exist without agency: (1) reflex pathways that incorporate conscious sensations as an intrinsic component (InCs), and (2) reflexes that are consciously conditioned and dependent on synaptic plasticity but not memory (CCRs). Whether InCs and CCRs exist as more than hypothetical constructs is not clear, and InCs are in any case limited to theories where consciousness depends directly on EM field-based effects. Consciousness with agency, as we experience it, then belongs in a third category that allows for deliberate choice of alternative actions (DCs), where the key difference between this and CCR-level pathways is that DCs require access to explicit memory systems whereas CCRs do not. CCRs are nevertheless useful from a heuristic standpoint as a conceptual model for how conscious inputs could act to refine routine behaviors while allowing evolution to optimize phenomenal experience (i.e., qualia) in the absence of individual agency, a somewhat counterintuitive result. However, so long as CCRs are not a required precondition for the evolution of memory-dependent DC-level processes, the later could have evolved first. If so, the adaptive benefit of consciousness when it first evolved may be linked as much to the role it plays in encoding memories as to any other function. The possibility that CCRs are more than a theoretical construct, and have played a role in the evolution of consciousness, argues against theories of consciousness focussed exclusively on higher-order functions as the appropriate way to deal with consciousness as it first evolved, as it develops in the early postnatal period of life, or with the conscious experiences of animals other than ourselves. An evolutionary perspective also resolves the problem of free will, that it is best treated as a property of a species rather than the individuals belonging to that species whereas, in contrast, agency is an attribute of individuals.

## 1 Introduction

This is one a series of papers designed to address the problem of how consciousness would have evolved, not to argue for specific scenarios, but to consider evolution as a process and how that process constrains what we can and cannot postulate to have occurred. The subject in this instance is mental causation, but approached through the lens of conscious agency, the role consciousness plays in directing individual actions. In dealing with how this may have changed with evolution, the more general issue of animal consciousness must be addressed, hence the relevance of the analysis to this Frontiers topic. The premise throughout is that consciousness of one kind or another is shared across a range of vertebrate taxa other than just our own. Most who take this view would accept any vertebrate with a neocortex or its equivalent as potentially hosting some form of consciousness, which would make it a shared feature of mammals and birds with origins somewhere among the reptiles (Cabanac et al., 2009; Allen and Trestman, 2020; Nieder, 2021; Tomasello, 2022), though a case can also be made for an earlier origin among anamniote vertebrates (Zacks and Jablonka, 2023). The behavioral evidence cited to support the neocortical premise rests on identifying in other species activities that closely resemble ones that for us would either be accompanied by conscious sensations or directly controlled by conscious decisions (Griffen and Speck, 2004; Birch et al., 2020a). Among these are the ability to communicate with conspecifics and engage in play and complex social interactions (Griffen, 1995; Preston and de Waal, 2002; Edelman et al., 2005; Cabanac et al., 2009), behavioral flexibility more generally, including planning for future events, problem-solving and engaging in deception (Birch et al., 2020a; Irwin, 2020; Kaufmann, 2021), expressing emotion (Panksepp, 1998; Panksepp, 2005; Bekoff, 2007), and having dreams with cognitive content (Hobson and Friston, 2012; Van De Poll and van Swinderen, 2021; Peña-Guzmán, 2022). With all such activities we face the problem that the presence of conscious experience in species other than our own cannot be proven, which raises the question of whether the study of animal consciousness can be considered science. To dispose of this objection at the outset, I would argue the opposite, that given the similarities our species shares with other amniote vertebrates in terms of brain structure and behavior, it would be poor science indeed not to accept the likelihood that some form of consciousness is also shared across those taxa. It is true that we cannot “visit” the brains of other species to determine with certainty that they too are capable of subjective experiences, but neither could Newton visit the moon and planets when developing his ideas on celestial mechanics so as to determine at first hand whether masses there were subject to gravity in the same way as they are on earth. Absence of certainty has not, in other words, been an impediment to scientific investigation and discovery in the past, nor should it be so today.

This account extends an evolutionary argument developed in a previous paper (Lacalli, 2023) that yielded two conclusions, most easily demonstrated for the subset of theories dependent on electromagnetic (EM) field effects: (1) that the events responsible for agency as a component of an evolving consciousness and those governing the character of phenomenal experience are separable and can be investigated as such, and (2) that it is possible to evolve a form of consciousness that is not epiphenomenal, but nevertheless fails to confer agency at the level of the individual. Using these results as a starting point, my intent here is to explore the concept of agency further to better understand the relationship between consciousness and agency and how that relationship could, in principle, have changed as consciousness evolved. My focus is on phenomenal contents as the best available markers for causal effect in an evolutionary context: that if evolution has assigned a sensation with distinctive characteristics to a particular sensory modality, it is a clear indication that the sensation was able to exert a causal influence on behavior at some point in the evolutionary sequence regardless of how this was achieved in mechanistic terms.

My analysis is developed through a series of thought experiments and arguments in principle, the premise being that anything possible in principle needs to be considered, but also that consciousness as it is today may conceal features that are best understood as relicts of consciousness as it was in the past. This applies regardless of theoretical stance so long as one is willing to accept, within the framework of a given theory, that the contents of consciousness are allowed to change over evolutionary time. Based on the pattern seen elsewhere in evolution, this would imply a trajectory from simple beginnings to something more complex meaning, for consciousness, that consciousness would today have more contents than it began with integrated in more complex ways. Ideally one would then want to be able to investigate the contents of consciousness individually in turn for evidence as to the proximate reason each evolved, its place in the sequence by which consciousness was elaborated and expanded by evolution, and for phenomenal contents, the reason the quale specific to that content was selected to represent it. So, for example, consider the global workspace in an animal with a newly evolved consciousness consisting of only two phenomenal contents. How would these be experienced, and how might that change as a third sensory modality was added to the mix? While such a question is, on the surface, highly speculative, seeking an answer is at core an exercise in thinking about whether all the contents of consciousness are there for the same reason or for different ones, and whether past additions to consciousness and the selection of suitable qualia to represent them has depended on the same mechanism each time or different ones. It is relevant then to consider how many such mechanisms might there be, where some theories may specify one, while others allow for more than one which, from my perspective, means there is something there that requires further investigation.

Point (2) above, relating to epiphenomenalism, figures in the story because of the long history of this issue in philosophical discourse (Robinson, 2023; see Lindahl, 1997 for the evolutionary aspects). There are ways to resolve it for a subset of theories of consciousness, specifically those depending on EM field-based effects, a result that I explore further both for its implications and to better understand the limitations of a neurophysical, EM field-based approach. From there I examine in some detail the process of action selection by biological brains, narrowing the focus further by treating agency not as any ability to act as the proximate cause of physical action, the way it enters into more general accounts of agency and causality (Jaeger, 2021; Potter and Mitchell, 2022; Ball, 2023), but instead as the ability to intervene in a sequence of non-conscious reflexive actions to alter that sequence. Three separate ways that conscious sensations could, in principle, influence behavior in this way can then be identified. These form a sequence of progressively increasing ability by the individual first to adjust, and then to control its own behavior. Whether this sequence actually occurred in the past, as part of the evolution of vertebrate consciousness, is less important than its heuristic utility, as a device for clarifying the relation between the ability to host conscious experiences and an individual’s control over its own actions. Two of the three categories fall short with regard to the latter, as in effect they are essentially reflexes, but ones where conscious inputs exert a modulating effect that alters the reflexive action in specific ways without directly controlling it. The third category, full agency at the individual level, would appear to require a learning processes and real-time access to specific memories of past events, so as to allow the individual to make an informed choice between alternative actions. In passing I refer at several points to Libet’s work on timing of intentional actions to illustrate an observational problem, that the integration of different forms of agency in our own consciousness may be so familiar to us as to conceal the complexity of what the conscious control of action actually entails. Yet, it is only by deconstructing the processes responsible that plausible hypotheses can be framed concerning the conscious experiences of species other than our own. A case study is included to illustrate this point, and I leave it to the reader to judge its value as a scientific exercise. The question of free will is taken up in the Conclusions, where I suggest an evolutionary way of framing the issue that eliminates the philosophical difficulties usually associated with it.

## 2 An evolutionary thought experiment: escaping the epiphenomenal trap

Given that neural activity is the proximate controller of behavior, there is a problem in explaining how consciously perceived sensations can be anything more than byproducts of that activity with no ability to effect behavior in and of themselves. This problem, of epiphenomenalism, has a long history as a philosophical conundrum, not only for those taking a dualist position, that the mind and body are separate, but also for a monist, materialist stance (Robinson, 2023). Epiphenomenalism is a particular problem for evolution because, absent a direct influence on behavior, there is no way for the contents of an emerging consciousness to be shaped by natural selection so as to be optimized for the functions they are required to perform. The evolutionary view is that emerging consciousness *must* be more than an epiphenomenon (Lindahl, 1997), but this only begs the question of how a consciousness conferring agency on the individual is acquired. If the potential for agency is there, waiting only for evolution to extract it, we still need to know what innovations at the neurocircuitry level are required to achieve this outcome.

**Figure 1:**
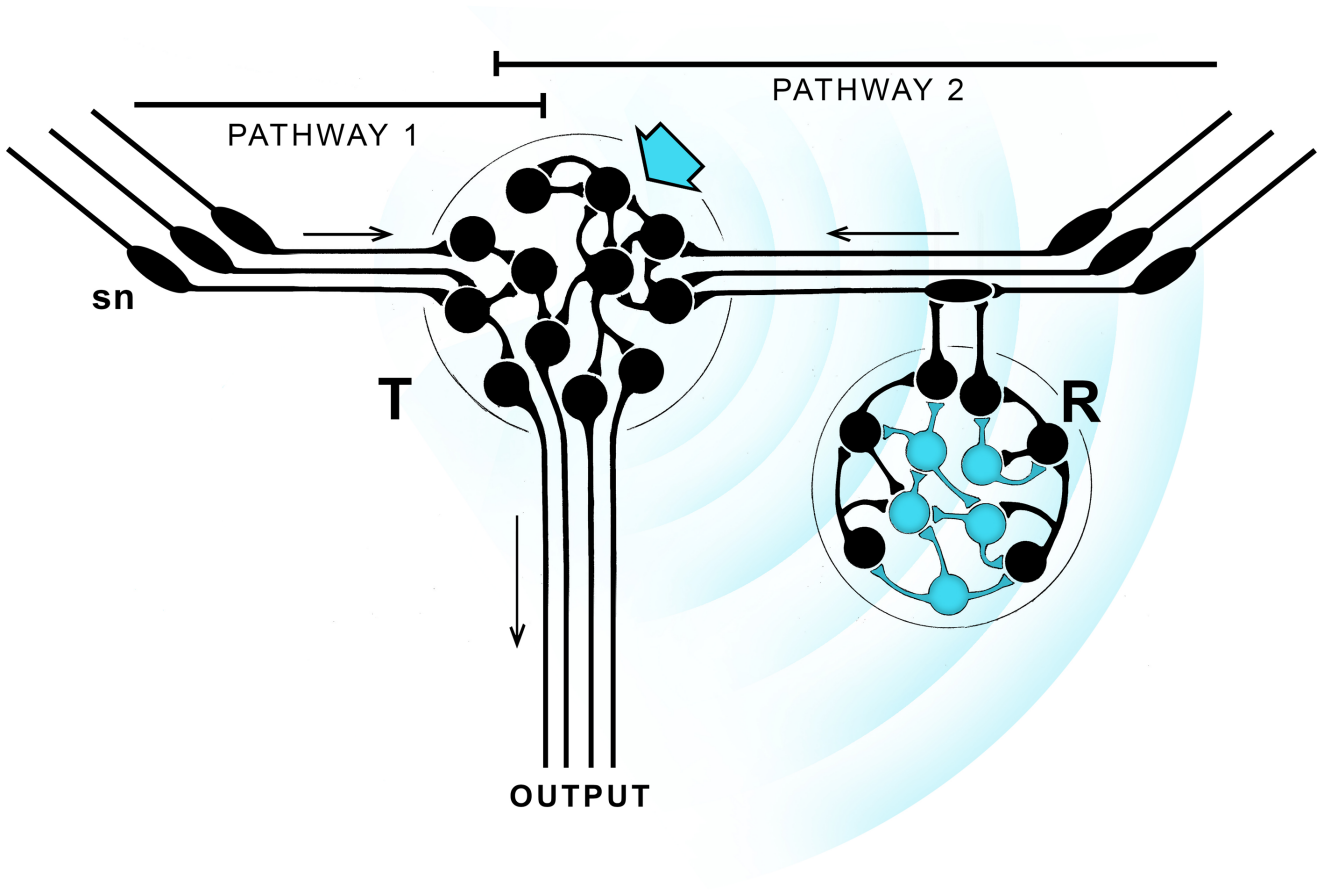
A hypothetical neurocircuit designed to illustrate how a pathway modulated by EM field-based extra-connectomal effects, whether consciously perceived of otherwise, could come to dominate over parallel pathways with no such modulation. The exercise repeats one in a previous paper, see Lacalli (2023), from which the figure is modified, for details. The starting point is two parallel pathways (1 and 2) with similar output, one modulated by EM field effects (blue waves) transmitted from one neuronal cluster (T, blue arrow) to another (R, blue neurons) that responds to it by modulating pathway 2, either by reinforcing or suppressing it in comparison with pathway 1. If the effect is a reinforcement that proves adaptive, then from generation to generation natural selection can refine and strengthen the effect until pathway 1 is rendered essentially irrelevant. If the signal is also one that is consciously perceived, conscious sensations will then be an integral component (InC) of pathway 2. But there is no individual agency here, as the diagram provides no route by which the individual can monitor the changing nature of the experience and act on it accordingly. Only the balance between pathways is changed, and that has occurred in evolutionary time, from generation to generation, not in real time. The result is a conscious pathway, essentially reflex-like, where the conscious component is more than an epiphenomenon, but the individual lacks agency, meaning the ability to adjust its behavior in real time in response to a consciously perceived sensation. Hence the qualitative character of the sensation, i.e., how it is “felt” by the individual, is irrelevant.

Hence the following thought experiment, which is explicitly neurophysical in assuming that consciousness is in some way a consequence of EM field-based phenomena. This is equivalent to a neuroscientific stance (Winters, 2021) and dependence on some aspect of “the physical” (Godfrey-Smith, 2019), but EM field-based effects are most commonly what such terms refer to (Hales and Ericson, 2022; Kitchener and Hales, 2022). Neurophysical theories, as a category, are well established as a way of dealing with the phenomenon of consciousness, but represent only a subset of all possible theories of consciousness (Atkinson et al., 2000; Seth and Bayne, 2022). A neurophysical stance is, however, the one that best suits my thought experiment, which I repeat here in abbreviated form from a previous paper, along with the relevant figure (Figure 1, modified from the figure in section 4 of Lacalli, 2023). Using fields or ideas derived from the physics of fields is widespread in the consciousness literature (Lindahl and Århem, 2016), in some instances simply as a conceptual device. I will be more explicit in supposing that these are real physical fields. The reason for dwelling here on a result specific to this one subset of theories is not, however, to advance the case for those theories over others, but is simply a device for clarifying the issues involved. This admittedly glosses over a number of problems, given the variety of ways EM field effects impinge on neural function and the difficulty of understanding what this means in causal terms (van Bree et al., 2024).

Figure 1 shows two parallel sensorimotor pathways one of which (pathway 2) incorporates neurons able to generate an EM field-based signal, indicated by the radiating blue waves. Unlike a signal broadcast across empty space, this depends on local fields produced by the neurons packing the intervening space, a process referred to as ephaptic coupling (Weiss and Faber, 2010; Hales and Ericson, 2022, Appendix A). There is then, in effect, an extra-connectomal component to neural activity, which means that even a detailed knowledge of the connectome would not fully account for the dynamics and output of the system.^1^ Ephaptic coupling could be a widespread modulator of brain function, or it may not. We do not know. Nor is it clear how neurons respond to such effects given the various ways this could, in principle, occur (Pinotsis et al., 2023). But for any brain region where local EM field effects are important, and given sufficiently high current densities, there is an opportunity for evolution to refine and adapt these for specific functions. The figure illustrates how this might be done, starting with two parallel pathways with similar output, one (pathway 2 in this case) modulated by an ephaptic signal while the other (pathway 1) is not. The modulation is accomplished by having neurons able to act as both signal transmitters (T) and responders (R). The figure shows these as being separate for purposes of illustration, but they could instead be integrated together in various ways, which is perhaps more likely given the limited spatial range of such effects. The point of the argument is that, under circumstances where the modulated pathway is more adaptive, natural selection will, over a series of generations, favor that pathway over the non-modulated one, while optimizing the signal to ensure that pathway 2 dominates. This would make the extra-connectomal EM field effect an intrinsic component (InC) of that now-dominant pathway.

A useful way to understand what has happened is to think of these effects in terms of waveforms representing solutions to the EM field equations, and visualize them as summations of different harmonic terms. Optimizing the signal then selects one waveform or set of waveforms over other possible solutions to the field equations. Unlike the propagated waves of synaptic activity monitored by conventional recording methods, this is more likely to involve complex and highly heterogeneous local fields generated by the sum total of neuronal activity measured at each point in 3D space (Jones, 2019; MacIver, 2022; Ward and Guevara, 2022), possibly including resonance effects of various kinds (Hunt and Schooler, 2019), all shaped by the boundary conditions imposed at each point in space by the structure and activity patterns of the neural substrate (Gómez-Emilsson and Percy, 2023). But so far, nothing has been said here about consciousness in relation to such field effects. In order to include a conscious component, one must suppose, in addition, that among all possible waveforms there are some that are capable of being perceived by a suitably adapted set of neurons in a way that results in a conscious sensation. If so, the pathway, though still a reflex, now has an intrinsic conscious component. From this point in the narrative “InC” will be used specifically to refer to pathways of this kind, which are then conscious reflexes in a sense, despite the ambiguity inherent in using the term reflex in this context. This does not then confer conscious agency on the individual because the change in neural output, and hence in behavior, from being controlled by pathways 1 and 2 in combination to control by pathway 2 alone, has been achieved by natural selection acting over evolutionary time, across generations, rather than through real-time decisions taken consciously by an individual. In other words, this is an entirely different situation from one where conscious control might be exerted intentionally over an otherwise automatic, non-conscious reflex, which would require that the individual is already endowed with agency over its own actions. Here, in contrast, that ability would not yet have evolved. It is then important to note that, for InCs, the waveform that optimally activates the modulated pathway will do so regardless of how the conscious sensation it represents is perceived by that individual. Even distinguishability may not matter for multiple such pathways representing different sensory modalities, since all could use the same sensation so long as the neurons involved are physically distant enough from one another that the signals they respond to do not interfere.

An InC is then a conscious pathway that is more than epiphenomenal, but leaves the individual deficient in agency. Two issues then need further clarification. The first relates to agency, and how that term is to be defined. Consider first an animal whose behavior consists entirely of non-conscious reflexes. Physical actions occur, and we can say that the animal is the proximate agent of those actions (Ball, 2023), in other words, an agent of action. But since reflex actions are preprogrammed to run their course once initiated, the individual is unable to intervene, and hence has no role as an agent of change. This, for the present account, is the key distinction, because it is only by identifying the agent of change that agency can be followed across an evolutionary sequence. An InC-level reflex, where the agent of change is evolution acting over multiple generations, is then very different from our own condition, that as individuals we have the ability to consciously alter our own behavior intentionally in real time. It is the transition between these two states that I wish to examine in the sections that follow, meaning the steps by which agency, in the sense of agency of change, might be transferred from evolution to the individual. InCs are then useful as a device for thinking about how that process may have begun.

The second issue is whether this thought experiment deals fully with the problem of epiphenomenalism or not. On the one hand it would appear to do so as long as one accepts that consciousness resides in the field itself or some aspect of its dynamics. EM fields may be invisible and somewhat mysterious, but they are not immaterial in the sense of residing somewhere other than real 3-dimensional space, and they have observable effects on material objects. The example above exploits this in supposing that it is the structure of the field that acts as the source of conscious sensations. One might think here of the sensations arising from a sensory noise field generated locally by the neural substrate, analogous to the noise of a radio signal, from which a signal, meaning a subcomponent of the field, could be extracted by having evolution arrange for some waveforms to be selectively amplified at the expense of others. At the core of this argument is an identity [e.g., see Lindahl (1997) and Jones and Hunt (2023)], that the waveforms associated with a particular solution to the field equations can be identified with a corresponding conscious sensation. This yields what I would call a “strong” form of EM field theory, equivalent to the reductionist category of theories discussed by Jones and Hunt (2023) in relation to ideas developed, most notably, by Pockett (2000, 2012).

The reductionist (i.e., strong) view then justifies the use I have made in this section of waveforms as a way of thinking about EM field-based effects. There is a downside to the identity concept, however, in that invoking identity to avoid epiphenomenalism fails to address the question posed by Velmans (2012; see also Chalmers, 1995), as to why any such pathway should be conscious when the same outcome could be achieved by a brain operating “in the dark.” This is because, assuming there may be extra-connectomal EM field effects that are not or cannot be perceived consciously, there is no reason in principle that those, as waveforms, could not be just as effective as a consciously perceived signal for activating InCs. A counterargument is that it might be that all EM field effects above a particular threshold in current density are experienced consciously, so there is no other option. Or, one could focus, not on the source of the signal, but its reception by neurons capable of responding to it (the Rs in the figure). What it means to be responsive to EM field effects and thereby cause a particular sensation to manifest itself is not known, and cannot even be said at this stage to be a fully formulated scientific question, but there is an option here for the response step in the process to be the point at which conscious pathways have the advantage over non-conscious ones.

A focus on the responding neurons then points to an alternative way of thinking about EM field effects, that instead of acting in effect as a source of conscious sensations, they do no more than stimulate receptive neurons to initiate a series of secondary processes, and those, by unspecified means, generate sensations of diverse kinds. Because this is simply a conjecture with no details attached, it is hard to argue either way that such processes would be epiphenomenal or not, and if not, which is what evolution requires, why that should be. Hence, among the various possible EM field theories, there should be “weak” variants where the identity does not apply, leaving the problem of epiphenomenalism unresolved. One needs also then to consider the many non-neurophysical theories competing with neurophysical ones to explain consciousness. This would include computational, representational, and functionalist theories, which one would like to be able assess in a systematic way for how they deal or fail to deal with foundational issues like epiphenomenalism. The task is complicated by different theories having different explanatory targets (Doerig et al., 2021; Kitchener and Hales, 2022; Jones and Hunt, 2023), so foundational issues are not always addressed, nor is it clear that they necessarily should be in all cases. This makes it hard to generalize about non-neurophysical theories, which are in consequence given less attention here than perhaps they deserve. I have little to add in any case to the summary provided by Paul et al. (2020) as to how the main categories of theory, as currently formulated, might apply to species other than our own.

## 3 Deconstructing agency: InCs, CCRs, and DCs

The preceding section develops an argument about InC level consciousness conceived of as arising from EM field effects. What follows is more general, in addressing questions that could potentially apply to any theory of consciousness requiring a learning process for the acquisition of agency by the individual. This accords with a more general (i.e., theory-independent) stance taken by some in the field, that consciousness (conscious agency in this formulation) must be learned (Cleeremans, 2011; Cleeremans et al., 2020; see also Jablonka and Ginsburg, 2022), or similarly, for theories where consciousness depends on the emergence of a self, that selfhood (agency implied) must be learned and achieved (Marchetti, 2022, 2024). Here I am not so much concerned with consciousness *per se*, but with conscious agency which, from my analysis is separable from other problematic issues relating to consciousness, including the hard problem and the nature of phenomenal experience (Lacalli, 2023), and requires a real-time interactive component for agency to reside fully with the individual. If this interactive process is equated with learning, in accord with Cleeremans, then we need only focus on how agency is learned, which means paying attention to different forms of learning. The distinction I make here is between simple and complex learning processes where, for the former, I would single out forms of conditioning that can occur without accessing stored memory.

**Figure 2:**
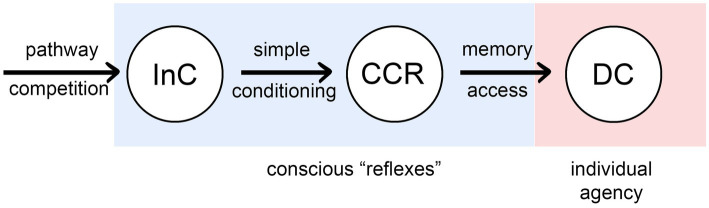
Three ways that consciousness could, in principle, exert an influence on behavioral control: as intrinsic components of reflex pathway (InCs) as in Figure 1, via consciously conditioned reflexes (CCRs), and through memory-dependent pathways that enable deliberate choices to be made (DCs). The first two (the conscious “reflexes”) are essentially reflex pathways that have been modified by conscious inputs, but differ in whether the effect occurs in evolutionary time across generations (InCs), or in developmental time as the brain and behavior develop (CCRs). DC dependence on memory, for species lacking language, would presumably involve visual, olfactory and mechanosensory records of past events, which raises the question of how these various types of conscious experience would be encoded, and whether the qualia involved would in some cases optimized for this purpose rather than some other.

Figure 2 summarizes the result of my analysis as a progression, left to right, of processes that confer increasing agency on the individual, from none (for InCs), to full agency as we would understand it, meaning the ability to consciously alter behavior through deliberate choice (DCs).^2^ The intermediate condition, as with InCs, is unproven but possible in principle, and so important as a point of theory: of consciously conditioned reflexes (CCRs) that are, as the name implies, reflexes, but ones modified by the conscious life experiences of the individual through a conditioning process dependent on synaptic plasticity and feedback, but not memory. CCRs allow behavioral actions to be adjusted and refined during development, and hence more rapidly than the generational timeframe over which InCs are adjusted and refined, but more slowly than behavioral changes under the control of DC-level neural pathways. CCRs could be considered to belong with other mechanisms for refining neural output through conditioning, even if these occur without direct conscious awareness, making them a subcategory of implicit (i.e., procedural) memory (Packard and Poldrack, 2003; Squire, 2004). I am being more restrictive, however, in defining CCRs as not depending at all on memory systems that store specific information on past sensory events. Only then is the causal structure of what is happening clear, that even without agency, inputs to CCR-level processes can exert a causal influence on the way conscious experience evolves, which means that the character of the experience as perceived by the individual will now matter in a way that it did not with InCs.

So consider in mechanistic terms how a CCR might work. We must first assume the animal in question is capable of conscious sensations that are not epiphenomenal and so can affect neural activity, through EM field effects for a neurophysical formulation, but by other means, unspecified, for other kinds of theories. The model I will use, the simplest available that will do the job, is synaptic pruning. Synaptic pruning removes excess synaptic connections through activity-based feedback, so that less active synapses are preferentially eliminated (Low and Cheng, 2006; Riccomagno and Kolodnik, 2015). This, coupled with the stabilizing effects of long-term potentiation, would appear to account for a significant amount of the restructuring of cortical synaptic networks that occurs during early postnatal brain development (Petanjek et al., 2023). It serves then as a minimal model for learning in the absence of memory access. To put this in a behavioral context, consider a hatchling engaged in a bout of activity in the nest, perhaps an altricial bird that has only recently hatched and whose memory systems we might suppose to be still developing and less than fully functional. Suppose some of its actions result in a sensation of pain or negative affect (e.g., anxiety or discomfort), which could be from contact with hard objects, or perhaps from being pecked by disturbed nest-mates. The pain/affect sensations exist because we are assuming the species hosts consciousness, and that the circuits responsible for those sensations are by this stage functional. We might then observe, that after several vigorous bouts of activity resulting in a painful or discomforting result, those bouts decrease in frequency and vigor. This could be achieved by conditioning without memory as follows: suppose the bouts of activity consist of a randomly selected assortment of gestures, some too vigorous and/or inappropriate for the situation, others better suited to it. Suppose also the conscious sensation generated by the less suitable subset of gestures, i.e., pain or discomfort, has the effect of suppressing any further activity for a period of time. Bouts where a greater fraction of gestures than average are of an unsuitable kind will then be suppressed more rapidly and for longer than those with a lesser fraction of such gestures, and consequently the synapses responsible for unsuitable gestures will, on average, be less active over a given time frame than those responsible for more suitable actions and will be preferentially eliminated as the pruning process proceeds. Across evolutionary time, assuming the adaptive benefit the mechanism is enhanced the more rapidly it occurs, selection will favor the conscious inputs (i.e., sensations) that are most effective both for suppressing unsuitable behaviors in the moment and for prolonging the effect to promote pruning.

This, for various theories, would provide the explanation for the particular character of any sensation that evolved specifically to facilitate CCR-type conditioning. Pain for example, would then be “painful” because it is the sensation that most effectively facilitates the process by which avoidance behaviors are learned and refined at a CCR level. If some other quale had proven more effective at this task, say the smell of a rose, then that is how pain would be experienced. By adopting a strong neurophysical, EM field-based stance, one could go somewhat further in terms of visualizing the waveforms that might be involved, e.g., that motor actions would be suppressed most effectively by waveforms of high amplitude (i.e., high intensity) rather than low, so as to dominate over competing sensations, but also that frequency range of the sensation should be narrow since, where the total amplitude is a sum of few components each of large amplitude, the decay time to the threshold below which the sensation is no longer perceived should be longer than for a sensation composed of many components with correspondingly lower amplitudes. Thus a “sharp” experience, as in sharp pain, should be more effective than its duller counterpart, meaning one that has a broader frequency range, which would likely preclude the smell of a rose from acting as an effective avoidance signal.

Regardless of specifics, the point I wish to make is a general one for agency at the CCR level regardless of theoretical stance: that the character of the experience as perceived by the individual now matters, where it did not for InCs. And, where the learning process simultaneously involves more than one sensory modality, it would matter that each signal is unambiguously associated with the modality it represents and no other, so that, for example, if pain is used as a signal for refining gestural actions, it will be distinguishable from visual or sound experiences whose perception might otherwise interfere. At the experimental level there is the problem of demonstrating the existence of CCRs as a category of circuits separate from other forms of implicit memory. But it is difficult in any case to identify the roles different forms of memory play in tests of cognition and behavior (Clark and Squire, 2007), while it is sufficient for my purposes that however important CCRs may or may not be to brain development, they are the least confusing way to clarify the issues that concern me here.

Agency as conferred by CCRs would appear to fall short of what we think of as acting with conscious intent. What we have instead is more like a reflex, but a conscious one in the limited sense that the reflexive action has been altered in specific ways, refined in other words, through the modulating effects of conscious sensations on past repetitions. In consequence, the action now unfolds in a different way once initiated than it would have otherwise. This provides a way for conscious sensations experienced in the past to influence behavior going forward on a continuing basis, but without requiring the recall of specific details of past events. It is then a more limited, less information-rich way of encoding past experience than is possible using a dedicated memory system, whether implicit, explicit, conscious, or not. Do CCR-level pathways then confer agency on the individual? The answer here is somewhat elusive. The fraction of agency attributable to evolution would appear to have decreased, because evolution has less direct influence over exactly what actions are performed in any given circumstance. In consequence the responses of different individuals would vary depending on how in each case their post-natal encounters with the external world have reshaped their brain circuitry. At the same time, the actions involved are still reflexes that the individual is not controlling intentionally, but are simply bouts of activity initiated by particular stimuli and accompanied by sensations of particular kinds. This begs a question that is potentially interesting in relation to broader issues of causality and the ontological structure of reality: whether agency, like some other physical phenomena, obeys a conservation law. If so, a reduction of agency in one part of a system would be compensated by a corresponding increase elsewhere. I have no answer here except to note that in the transition from InCs to CCRs, while there is no obvious increase in agency for the individual, there could be for some combined entity consisting of the individual and that individual’s real-world experiences. But this still falls short of what we expect of agency acting at the level of DCs.

**Figure 3:**
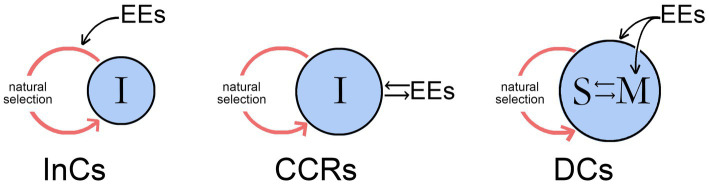
The three modes of behavioral control from Figure 2 showing how in each case time scale enters into the action system consisting of an evolving individual (I) and its environment where external events (EEs) impinge on the system in different ways. For InCs, the EEs act through natural selection (the red loop in each case) to affect the way the conscious signal evolves, but despite the individual being the proximate agent of action, it is otherwise a passive participant in the process. For CCRs, the individual has an active relationship with the EEs in real time, during brain development and possibly later on an ongoing basis, an option that allows the contents of its consciousness to evolve according to how they are perceived by the individual but without conferring agency. DCs do confer agency, and differ from the previous two categories in that the individual now stores information on the conscious sensations associated with past events in memory (M) and possesses a mechanism, here represented as incorporating a self-like entity (S) for accessing and assessing that information. EEs in this case have inputs to both memory and the self, but the interaction, which was for CCRs external to the individual and operated in the time scale of that interaction, is now internalized and proceeds at the speed of neurophysiological events. It is this feature, as well as the ability to compare past and present in real time, that is the defining feature of DCs, distinguishing them from CCRs, where the past is always and only in the past.

Since CCR-level pathways fail to confer agency on the individual, they would not be involved in the control of neural responses incorporating conscious planning and deliberate choice between options, which instead would require DC-level pathways. With DCs we are on firmer ground than with CCRs, because DC-level agency is what we expect of consciousness based on our own experience. For DCs to incorporate pathways dependent on the recall of past conscious experiences, access to specific memories is required however those memories are encoded. Agency is not a problem if we can assume, for whatever theoretical stance has been adopted, that conscious sensations are not epiphenomenal. Non-conscious sensorimotor pathways that incorporate memory could then simply add a conscious input. The puzzle that remains is instead how this would be accomplished at a neurocircuitry level, which could be through alterations to the memory circuits themselves or through links established to separate non-memory systems. Few theories of consciousness concern themselves with the details here, or with deconstructing agency as I have done. Most, in fact, would probably accept DC-level processes as the only ones that need to be considered. Why then bother with CCRs at all, or InCs for that matter, if they fall outside the concerns of a considerable body of theory? One answer is that, not knowing in advance which theories or categories of theory are correct, it is better if our working list of what needs explanation is as complete as possible, even if proponents of different theories disagree on what that list should contain. For CCRs in particular, perhaps a better answer is that between them, CCRs and DCs represent two quite different ways the individual and the external world can interact. As shown in Figure 3, for CCRs the interaction is in real time between external events and the individual, but the effects are developmental and cumulative, so the ability of the individual to make significant real-time changes to its own behavior is limited. DCs, through memory, allow the interaction with the residue of past events to occur internally, within the brain, and at a rate limited only by the speed at which memory is stored, accessed and assessed. Rapid and quite specific changes to behavior are then possible. What in my view then validates InCs, CCRs, and DCs as models for behavioral control is that between them they would appear to exhaust the possible ways that interactions between the individual, its brain, and external events can be accommodated within the available time scales, of evolutionary time, real time (for developmental changes), and the compressed time scale of neurophysiological events as this relates to memory function.

It is useful in this context to point out what CCRs cannot do, and which then by default must be a capability of DC-level processes. Here Velman’s question again becomes important, of why brains might not just as well operate without consciousness, in the dark. For CCRs, one would have to suppose that synaptic pruning responded differently to conscious versus non-conscious inputs. For DCs, the answer would instead relate more specifically to the way conscious inputs influence dedicated memory systems. This suggests at least the possibility that consciousness, as it first evolved, provided a new and better way of encoding sensory events in memory or of making subsequent use of that encoding. This could be because it provides an especially effective way of tagging memories for their phenomenal associations so as to facilitate their recall and later use, which means also that the sensations evoked may be optimized for this function rather than some other.

## 4 The question of sequence ─ evolutionary and developmental

The sequence shown in Figure 2 for InCs, CCRs, and DCs, is one of decreasing agency for evolution and increasing agency for the individual. Whether this maps to a real sequence, either during evolution or development, are separate questions, and there is the added problem of whether InCs and CCRs are more than hypothetical constructs. I am not prepared to argue the case for InCs because they are meaningful only for a subset of EM field-based theories. Within that context, however, and assuming InCs could be a precursor to more evolved forms of consciousness, they could be the form consciousness takes among some lower vertebrate taxa. CCRs are more interesting as a subject for speculation because they are relevant to a broader range of theories. And, whether they evolved before, after or concurrently with DCs, the different time scales on which CCRs and DCs operate would argue for both being subsequently retained and employed in a complementary way for different functions. Hence, even if CCRs evolved early as a component of a rudimentary form of consciousness, they could have been retained for the tasks for which they are best suited, including a developmental role in shaping and modulating behaviors that are habitual, repetitious and performed rapidly. Examples might be simple foraging actions such as scratching or pecking at the ground, where the conscious inputs might be from multiple sensory modalities, e.g., touch, vision and olfaction, but the behaviors are ones that can be carried out, like a reflex, with minimal intervention, at least until a particular food item is encountered that needs to be dealt with in a particular way. In contrast, there are events in an animal’s daily life that require access to specific memories and a deliberate and considered response. An example might be spotting a predator in a particular tree or shrub which needs both avoidance in the present time and a wide berth in future. There is a particularity to this situation, because it is not all such trees or shrubs that are to be avoided as a matter of habit, but only certain ones under certain circumstances. We are then in the realm of DC-type agency, which means a longer response time because information encoded in memory must be accessed and compared with current sensory inputs.

One might suppose that CCRs, being mechanistically simpler, would have evolved before DCs. But in fact the opposite might be true if most if not all of the memory systems required for DC-level functions evolved before the neocortex became a major center for sensory processing. This appears to be the case, given the presence of homologs of memory-related structures such as the amygdala and hippocampus in basal vertebrates (Zacks and Jablonka, 2023; Bingman et al., 2024). DC-level pathways could conceivably then have been the basis for consciousness right from the start. So the answer to Velman’s question could be that the adaptive value of consciousness relates to its role in memory function. This accords with the case made by Shea and Heyes (2009) for placing explicit memory at the center of investigations into animal consciousness, and also with the associative learning model of consciousness proposed by Ginsburg and Jablonka (Birch et al., 2020b; Jablonka and Ginsburg, 2022) whose evolutionary transition marker (ETM) I would expect to map to the CCR-to-DC transition. But the memory-based theory that is most interesting in the present context is that of Budson et al. (2022; see also Hogendoorn, 2023) because of the position those authors take on epiphenomenalism: that consciousness is epiphenomenal in relation to some functions, notably action selection, because of the time delay inherent in accessing memory pathways, but this cannot at the same time mean that consciousness is truly epiphenomenal when conscious sensations are required to materially affect memory. What this illustrates is a general point, that for any theory of consciousness where memory is a central component, there is a distinction to be made between the role of consciousness in initiating and directing actions, which may appear to be epiphenomenal in experimental tests like Libet’s, and epiphenomenalism as an ontological principle, meaning the question of whether and how conscious sensations affect neural function, specifically in this case in memory centers like the hippocampus.^3^ We would then be dealing with some form of synaptic plasticity responsive to consciously perceived inputs, which would almost certainly operate on a more rapid time scale than the CCR-level conditioning process I have described above.

With regard to the time delay inherent in neural pathways that involve memory access and recall, it is worth pointing out that, rather than a design flaw, this may simply be a relict of the way evolving consciousness was first integrated into vertebrate behavior. Consider, for example, an evolutionary scenario where emerging consciousness was used primarily to switch between approach and avoidance behavior based on how current sensory inputs alter the balance of positive and negative affect those inputs are linked to in memory. The individual animal will have a conscious sense of its motivational state at any point in time based on this balance that is updated on an ongoing basis. But because memory formation and access impose a time delay, there will be an interval between the point in time that a given sensation is experienced and the time the motivational state fully reflects the consequences, at the neurocircuitry level, of that new input. In that interval, actions initiated in direct response to the event producing the sensation may already have begun, and conscious inputs to those, in their role as part of the process of initiating the action, would reflect the earlier motivational state. This is a more tangible way of thinking about what a Budson et al. (2022) mean when they say (their pg. 270) that consciously we “perceive the world as a memory.” And, for behaviors where the sensory inputs that influence motivational state change over a longer time frame than the interval by which memory is updated, the system is adaptive, which should be the case for routine behaviors that unfold gradually. Experiments like Libet’s on the timing of cortical events and the actions with which they are associated can then be interpreted as revealing the limitations of the mechanism, which is what those experiments were designed to do in any case.

There is then the question of whether memory systems themselves follow an evolutionary sequence, so that, for example, if olfactory memory evolved before, say, visual memory, a conscious awareness of olfactory stimuli and the ability to respond accordingly would have evolved before its visual counterpart. Or vice versa. An early origin for both CCRs and DCs would also imply a long period of coevolution, which complicates the task of disentangling the respective contributions each makes to behavior, and to the development of a self, if indeed that is a precondition for DC-level consciousness [e.g., see Marchetti (2024)]. The difficulty of interpreting experiments like Libet’s (David et al., 2008; Neafsey, 2021; Triggiani et al., 2023) is not then surprising. Dreaming should also be considered in this context (Fazekas and Nemeth, 2018; Pantani et al., 2018) because it is necessarily, assuming the dream in question is a conscious one, a DC-level process. This would imply, from my analysis, that animals capable of conscious dreams fall into the same category as those with conscious agency, meaning for some species the former might provide a useful test for the latter.

Turning to the question of developmental sequence, the key point relates to infantile amnesia, a property of human development that we, as an altricial species, likely share with numerous other mammals. This is the period when the newborn shows evidence of phenomenal consciousness (Lagercrantz and Changeux, 2010; Bayne et al., 2023), but its memory systems are not yet fully developed (Hayne, 2004; Alberini and Travaglia, 2017). One view is that intentionality in the sense of goal-directed actions develops earlier, *in utero* (Delafield-Butt and Gangopadhyay, 2013; Ciaunica et al., 2021). But if memory systems are not fully functional until sometime after birth, there would necessarily be a transitional late fetal or early postnatal period when any refinement of motor function requiring conscious sensations as an input would have to be accomplished by some means other than DC-level pathways. The issue is then one of learning more about the development of memory systems across species in the pre- and post-natal/post-hatching period and how this relates in terms of timing to other events of brain maturation, including synaptic pruning. Provisionally, the conclusion I would draw from the human experience of infantile amnesia is that it is circumstantial evidence that postnatal brain maturation could incorporate CCR-type processes as an important component.

## 5 A case study: Tipper’s new harness

Ethology, the study of animal behavior, places little value on anecdotal observations unless they can be replicated under controlled conditions in a statistically significant way. The internal mental state of the individual subjects has also not, in the past, been a matter of concern, as it cannot be measured, nor have speculations on the subject been encouraged. As scientists we thus have something of a blank slate when it comes to the question of animal consciousness, where criteria for judging whether a given observation is significant or not as an indicator of a conscious mental state are still rather fluid. As models for how the question might be investigated, one could point to observations by Bates et al. (2008) on elephant empathy, Irwin (2024) at the Denver Zoo, or Pepperberg (1999) on her parrot Alex. The last is more of a case history approach and is the one I adopt here, the intent not being to prove anything specific about animal consciousness, but to show that the ideas developed in previous sections are not entirely at odds with observation.

**Figure 4:**
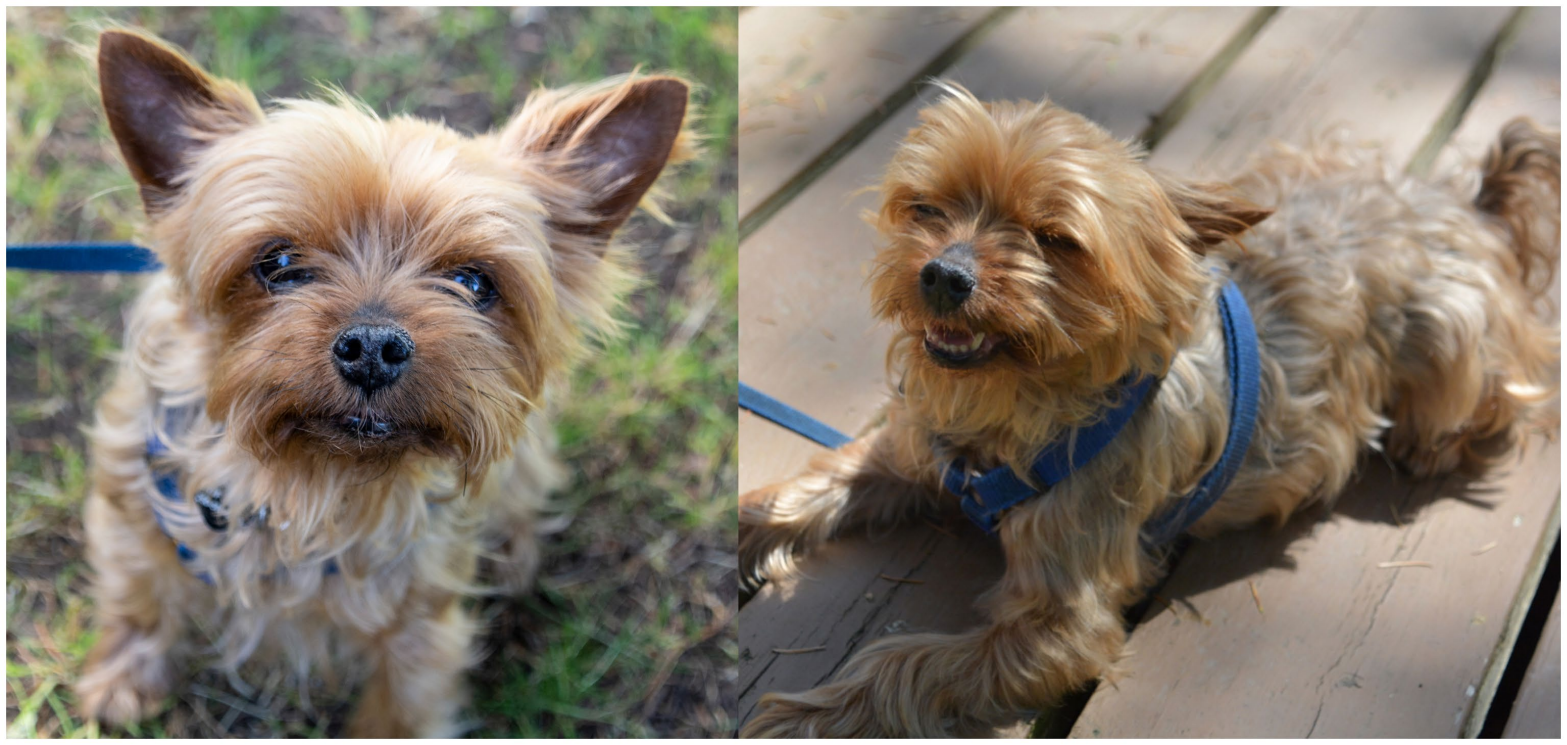
Tipper, shown here in her old harness. The learning process required to put on a new one, of a somewhat different design, shows, by its time course, that it could be explained by simple conditioning subject to conscious inputs. This does not prove the case, but provides justification for the emphasis given in this account to consciously conditioned reflexes (CCRs) as a way of adjusting and refining routine behaviors. Photo credit © M. McNay.

My subject is an alert and very lively 12 year-old Yorkshire Terrier named Tipper (Figure 4). Being too excitable to wander freely outdoors, she wears a harness, until recently one that goes first over her head, after which she must step forward through an opening between the straps with her left foreleg before the harness can be buckled across her back. A similar stepping motion is required when putting on her winter sweater, so stepping is a familiar action to her when either the harness or the sweater is presented. Having broken a clasp, the old harness was replaced recently with a new one that is fitted over each foreleg from below before the clasp can be closed. Stepping motions interfere with this, as instead what is required is for each foreleg in turn to be raised into a tucked position and held immobile while the harness is fitted. To do this, Tipper needed to learn to suppress stepping actions in favor of tucking, meaning either that she has to forget an established behavior lodged in memory, replacing it with a new memory, or, if this is a conditioning process, that the reflex involved must be reconditioned. The learning process for this transition required about 4 weeks of multiple trials each day, for a total of about 80 replicates before she was able for the first time to put on the harness smoothly, without extraneous actions. Initially she executed repeated stepping actions with each leg in turn. When these failed to complete the task, these actions were followed by a series of random waving gestures directed at various angles and heights. Among these, tucking was at first a rare event, but increased in frequency and duration, first as a late response, then an earlier one, while the frequency of stepping and waving actions correspondingly declined. The main cues as to the progress of each trial appear to be tactile rather than visual, since much of what is transpiring is outside her range of view, while watching the harness strongly increased the tendency for stepping, even after the training process was well advanced. As to evidence of her mental state during training, beyond an awareness of tactile sensations, negative affect is also apparent, of irritation or frustration, evidenced by actions that are increasingly erratic during trials requiring an extended period of time.

What then has caused this behavioral change? It could involve suppressing or forgetting explicit memories as mentioned, but simple conditioning also explains the observations and the extended time required, so the involvement of CCR-type pathways cannot be ruled out. The point of this case study is not, however, to resolve the issue of mechanism because, given the number of options, it cannot. Instead, I simply want to illustrate how the issues at stake change once the involvement of conscious sensations is provisionally accepted: that the focus is then on the intricacies of how memory systems are integrated with motor control, something that can be treated as a problem of conventional neuroscience and investigated accordingly. As I write this, Tipper is sitting on a dining room carpet staring dejectedly at a recently polished stretch of the floor across which she is unwilling to venture. Two trials of making the attempt and losing her footing now inhibit her from a further attempt. Her behavior in this case would appear almost certainly to depend on explicit memory, whether it involves hippocampal place cells or visual cues or both, encoding the conscious distress of scrabbling about on a slippery floor. This, compared with the harnessing exercise, is clearly a very different process, both phenomenologically and mechanistically. What remains to be determined is what precisely these differences are.

## 6 Conclusion, with a note on free will

Assuming, as I do here, that consciousness occurs across a range of vertebrate species, any theory that purports to be a theory *of consciousness*, and not simply of certain particulars of human consciousness, should apply equally to those other species. One can ask, for example, whether global workspace theory (GWT) should apply as much to the mental processes of a mouse while foraging as it does to our own conscious thoughts and actions, which begs the question of what, absent language, would act for the mouse as a common currency for encoding meaning. Paul et al. (2020) explore this issue at some length in their survey of various theories, and Zacks and Jablonka (2023) do so specifically in relation to GWT, but the onus should in my view be on proponents of each category of theory to provide definitive answers. Not to do so is to give insufficient weight to the evident similarity of brains and behavior across amniote vertebrates, which means more generally, that the issue of how consciousness would have been assembled by evolution acting over an extended period of time deserves more attention than it has so far received. This bears also on questions of the form “what it is like to be...?” for species other than our own. If we assume that the contents of phenomenal consciousness were originally adjusted and refined over an extended evolutionary timeframe so as to optimize them for particular functions, then so long as those functions are unchanged in later evolving taxa, the conscious experiences of those taxa should be minimally changed if at all.

The proximate reason for writing this paper is to explore more fully an argument made by Cleeremans (2011) that consciousness must be learned. My previous analysis of the issue (Lacalli, 2023) led me to conclude that this, in more precise terms, means that agency must be learned, so that consciousness of the kind we are familiar with, which confers agency at the level of the individual, requires a learning process repeated in each generation. To extend the analysis, different kinds of learning processes need to be examined to assess their ability to confer individual agency. The distinction I have made here is between learning processes involving simple conditioning but not memory storage and recall (CCRs), and those that depend on dedicated memory systems that do perform those functions (DCs), where it is only DCs that confer agency at the individual level. What is then a surprising and somewhat counterintuitive result is that CCRs, even without agency, are capable of driving the diversification and refinement of phenomenal contents over evolutionary time so as to optimize them for their role in the conditioning process. Individual agency is therefore not required, at least in principle, for a function it might have been expected to control: of being the only reason that distinguishable qualia have evolved in particular ways rather than others. The role CCRs play in this process in real brains as opposed to hypothetical ones has yet to be determined, but it could be an important one.

The distinction I have drawn in this analysis between CCR- and DC-level processes is not dissimilar to that between phenomenal (P-) and access (A-) consciousness according to Block (1995, 2005), with “access” equating to the recall of specific information lodged in or dependent on memory. Block’s ideas have since generated a substantial literature, revealing a number of conceptual difficulties (Schlicht, 2012; Overgaard, 2018; Wu, 2018). The approach I have taken is somewhat different and, I would argue, less problematic. To illustrate this, consider this quote, from Naccache (2018, p. 2) paraphrasing Block: that “we know we are phenomenally conscious because we access [the] experience and consciously report it.” But both knowing and reporting are complex neurobiological processes, drawing on memory, and on language in our species for the inner voice that informs us about our mental states, which then makes it difficult to disentangle what is happening in mechanistic terms (Halligan and Oakley, 2021). The CCR/DC distinction avoids these complications because, rather than approaching the problem from a phenomenological point of view, it instead uses a mechanistic difference to distinguish between two categories of neurobiological processes. Experimentally there is still the problem of assessing the respective roles of CCRs and DCs in controlling behavior when they operate together, which simply adds to the already difficult problem of interpreting experiments on the timing of cortical events in relation to the initiation of actions.

A final point concerns free will, a perennial concern for the philosophical community (O’Conner and Franklin, 2022). The problem here is that if the brain is responsible for all of behavior, whether consciously controlled or not, then as individuals we are prisoners of the choices our brains make, whatever those are and for whatever reason. What we then “choose” to do is what we would have done in any case, which negates the idea that any choice was in fact possible. From an evolutionary perspective, the solution to this conundrum is to cast the argument not at the level of the individual, but of the population. This is biologically the correct stance in that no individual exists except as a member of a population of individuals belonging to the same species linked by evolution to past members of that species. The distinction I would then make is between the term agency, which can be applied with caveats to individuals, as described above, and free will, which I would argue cannot. Instead, free will is better viewed as a measure of the range of behavior across a species, so that individual actions are variants on a norm. Species with free will would then be defined as those where this range is greater than can be accounted for by genetic and phenotypic variance, but instead reflects the varied life history experiences of diverse individuals and the way learning and memory have shaped each individual’s actions and decisions. In consequence, we can on the one hand accept that the actions of an individual are predetermined by circumstances and past events specific to that individual while, on the other, granting each individual free will by virtue of belonging to a species that can be shown by a suitable quantitative test at the population level to possess free will. It is a category error to do otherwise, which is not to deny that individuals belonging to a species with free will have greater freedom, as individuals, to behave differently in a given circumstance than conspecifics.

Free will considered as a species characteristic is then related to individual agency, but not identical to it. And, as a species characteristic that can, in principle be quantified, it allows species to be compared to determine whether some have more free will, or less, than others. I make this point in part to show how the philosophical question can be recast as a scientific one, but it should not be surprising that using evolutionary arguments to do so depends on changing the focus from the individual to the population and species, as evolution is fundamentally a population- and species-level phenomenon.

## Data availability statement

The original contributions presented in the study are included in the article, further inquiries can be directed to the corresponding author.

## Author contributions

TL: Conceptualization, Writing – original draft.

## Footnotes

^1^Paracrine neurotransmitter release, a separate category of extra-connectomal effects, has been left out of this account to avoid complicating the argument, but would have to be included in any full explanation of neural output.

^2^“Deliberate” is used here for two reasons, first because the choice is a deliberate one made by the individual, but also for the implied deliberative process, which would require a specific neural structure and a period of time for its execution.

^3^The “ghost in the machine” argument is in my view largely a matter of conflating these two targets of action.
